# Recent Advances in Marine-Derived Polysaccharide Hydrogels: Innovative Applications and Challenges in Emerging Food Fields

**DOI:** 10.3390/polym17182553

**Published:** 2025-09-21

**Authors:** Xinge Yi, Jing Xie, Jun Mei

**Affiliations:** 1 College of Food Science and Technology, Shanghai Ocean University, Shanghai 201306, China; 2 National Experimental Teaching Demonstration Center for Food Science and Engineering, Shanghai Ocean University, Shanghai 201306, China; 3 Shanghai Engineering Research Center of Aquatic Product Processing and Preservation, Shanghai Ocean University, Shanghai 201306, China; 4 Shanghai Professional Technology Service Platform on Cold Chain Equipment Performance and Energy Saving Evaluation, Shanghai Ocean University, Shanghai 201306, China

**Keywords:** blue foods, polysaccharides, biological activity, hydrogel, food applications

## Abstract

Marine-derived polysaccharides (MPs) are a class of polysaccharides isolated and purified from marine organisms, which engage in various biological activities such as immunomodulation, anti-tumor, antibacterial, antioxidant, and anticoagulant activities. Excellent biocompatibility, biodegradability, and low toxicity make them ideal biomaterials for the preparation of hydrogels. In recent years, MP-based hydrogels have been successfully fabricated into various novel and smart hydrogels, triggering new transformations in the fields of biomedicine, cosmetics, and food. This review introduces the structural features, bioactive mechanisms, and safety evaluation of MPs. This review focuses on the latest application progress of MP-based hydrogels in the food field, including fruits and vegetables, meat products, aquatic products, bakery products, and health products, aiming to provide fundamental support for further research and development in the food industry.

## 1. Introduction

Marine-derived polysaccharides (MPs) are macromolecules isolated and purified from marine or lake organisms. According to different sources, they are categorized into marine animal polysaccharides, marine plant polysaccharides, and marine microbial polysaccharides. Marine animal polysaccharides are mainly derived from invertebrates and fish. Based on different applications, MPs are categorized into gel-forming polysaccharides and bioactive polysaccharides [1]. Marine plant polysaccharides, especially algal polysaccharides, are among the richest natural resources of the oceans. They are commonly used as a gelling agent, stabilizer, or thickener in various industries such as confectionery, medicine, and dairy products [2,3]. Marine microbial polysaccharides are mainly extracellular polysaccharides with distinctive activities. However, very few species can be commercialized and put into production [4].

As they are among the most abundant substances in the ocean, the anti-obesity [5], anticoagulant [6], antibacterial [7], anti-inflammatory [8], and antioxidant [9] effects of MPs have been widely reported. Compared with terrestrial polysaccharides, MPs are unique in structure, physical properties, biocompatibility, and biological activities, having advantages in pharmacological stability, extraction, and purification processes [10]. This is mainly because marine organisms typically live in special environments with high pressure, high salinity, low temperatures, and low light intensity. Interactions between tissues and cells tend to be stronger than in terrestrial organisms [1]. In terms of structure, the main difference between MPs and others is the presence of sulfate and amide groups [1,3]. In terms of biological activity, MPs have lower toxic side effects and more effective activities [10].

Due to their excellent biocompatibility, biodegradability, and desirable properties such as low toxicity and multiple bioactivities, MPs have shown broad application prospects in biological materials [1,10,11,12]. With the application fields of MPs expanding, hydrogels, emerging as one of the multifunctional materials, have triggered great interest among researchers in various fields because of their unique characteristics. They consist of three-dimensional cross-linked polymer networks with excellent hydrophilic properties [13]. Due to the cross-linking present in the polymer chains, hydrogels can not only swell in water and absorb a considerable amount of water or biological fluids but also prevent themselves from dissolving [14]. MPs are ideal materials for the development of hydrogels. They contain abundant functional groups such as carboxyl, amino, hydroxyl, and sulfate groups. These functional groups can serve as active sites for chemical reactions, rendering MPs susceptible to functional modification and providing multiple possibilities for designing functional hydrogels. Chitosan (CS), hyaluronic acid (HA), cellulose, agar, carrageenan, and sodium alginate (SA) are frequently employed in the fabrication of hydrogels featuring various structures and properties. These hydrogels are widely applied in food fields such as food processing [15], food packaging [16], nutrient delivery [17], tissue engineering [18], sensors [19], and thermal insulation [20]. These properties confer MPs with unique advantages in hydrogel design. They can provide better options in terms of mechanical properties, safety, encapsulation rate, and thoughtful response compared to polysaccharides from terrestrial sources.

The number of research articles on MP-based hydrogels has been increasing over the past five years. Although the formation and delivery matrices of MP-based hydrogels have been reported in scientific literature, these works mainly focused on the biomedical field (e.g., drug delivery systems, wound dressings, and tissue engineering). Recent reviews have demonstrated that interactions between MPs and other components in the food system will inevitably take place in several ways, and these interactions may have a significant impact on the structure and properties of the foods. However, to date, research on the application of polysaccharide hydrogels in the food industry lags far behind that in the biomedical field. Moreover, the information on the application of MP-based hydrogels in food is scattered. Overall, there still exists a significant dearth of systematic descriptions and summaries concerning the characteristics and food applications of MP-based hydrogels. This article aims to bridge the existing knowledge gap by systematically reviewing and expanding on the latest trends and application boundaries of MPs in food.

The novelty of this review lies in the systematic summary of the key characteristics of MP-based hydrogels and their applications in various food sectors, including food packaging, food detection, nutrient encapsulation, and fat substitution, as well as the development of cultured meat and plant-based meat. Moreover, it dives into the structure-activity relationships governing the structure and biological activity of marine polysaccharides, while also examining their safety for food applications through in vivo and in vitro toxicity assessments. Finally, it mentions the potential challenges and prospects of MP-based hydrogels.

## 2. Review Methodology

This review was conducted through a systematic search of scientific databases, including Scopus, Web of Science, and ScienceDirect, focusing specially on peer-reviewed articles published from 2020 to 2025. The keyword combinations used in the search included terms such as “marine + polysaccharides,” “aquatic organisms + polysaccharides,” “algae + polysaccharides,” “algae + polysaccharides,” “chitosan + hydrogel,” “fucoidan + hydrogel,” “polysaccharides + hydrogel + packaging,” “polysaccharides + hydrogel + cultured meat,” and “polysaccharides + structure + biological activity,” among others.

The review focused on research articles presenting experimental data related to the hydrogel behavior of MPs, mainly within the context of food processing. Moreover, the bibliographies of retrieved citations were cross-referenced to achieve some additional articles. Exclusion criteria included studies confined to extraction methods and efficiency of MPs, the biological effects of the extracted polysaccharides without mechanistic analysis, or those concentrating on medical applications of hydrogels. The conference proceedings, abstracts, posters, and book chapters due to lack of peer review and review articles based on the recommended criteria were also excluded.

## 3. Structure of MPs

### 3.1. Monosaccharides and Backbone

**Figure 1:**
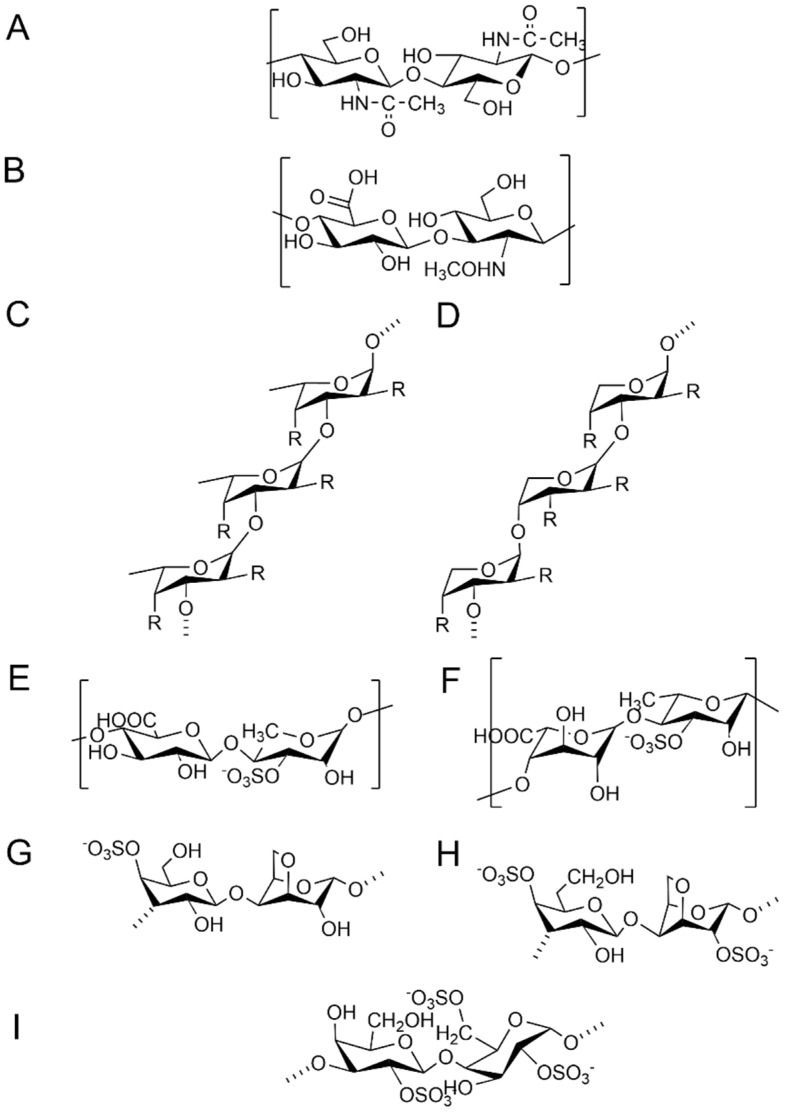
The backbone structure of chitin, hyaluronic acid, fucoidan, ulvan, and carrageenan. (**A**): Chitin. (**B**): Hyaluronic acid. (**C**): Type 1 fucoidan molecules. (**D**): Type 2 fucoidan molecules. (**E**): Ulvan type A_3s_. (**F**): Ulvan acid type B_3s_. (**G**): κ−carrageenan. (**H**): ι−carrageenan. (**I**): λ−carrageenan. (The R can be a monosaccharide or a sulfate group.).

The structural characteristics of MPs are first reflected in their monosaccharide composition and polysaccharide backbones. Common monosaccharides in MPs include glucose (Glc), galactose (Gal), mannose (Man), fucose (Fuc), and uronic acid [11]. The composition of MPs from different sources varies greatly. Fucoidan is mainly composed of fucose [21]. Agar and carrageenan are primarily composed of Gal. Glycosaminoglycans are primarily composed of alternating hexosamine and hexuronic acid (or Gal) [22]. Microbial polysaccharides, on the other hand, are composed of a variety of heteropolysaccharides [2]. Differences in the composition of monosaccharides directly affect the physical properties and biological activity of polysaccharides. For instance, disaccharide repeat units formed by glucuronic acid (GlcA) can promote the gelling properties of SA, while mannuronic acid units increase the viscosity of SA [23]. The carboxyl groups of uronic acids provide negative charges, serving as essential units for bioactive polysaccharides such as heparin and heparan sulfate [22]. In addition, polysaccharides with high uronic acid content promote phagocytosis by immune cells and stimulate cytokine production [24]. In terms of backbone structure, MPS can be categorized into four types: linear homopolymer structures (SA), periodic repetitions of disaccharide units on the polysaccharide backbone (glycosaminoglycans), complex branched structure (fucoidan), and irregular, non-periodic complex structure (containing multiple types of monosaccharides or adhering to proteins or lipids) [25]. There are differences in the kinds of glycosidic bonds in polysaccharides from different sources. The backbone structures of common MPs are shown in Figure 1. The 1→4 glycosidic bond is most common in MPs. α-(1,4)- and α-(1,6)-D-glucans are commonly found in the polysaccharides of green and red algal, whereas β-(1,3)-D-glucans are commonly present in brown algal [26].

### 3.2. Molecular Weight

Polysaccharides of the same type but with different MWs exhibit different biological activities. For example, HA of 6–20 kDa mainly exhibits immunomodulatory, angiogenic, and pro-apoptotic activities, while HA of 20–200 kDa can participate in wound healing. Higher MW HA (>500 kDa) has anti-angiogenic activity [27]. In general, LMW polysaccharides can show potent biological activities. This is mainly because polysaccharides with too high molecular weight (HMW) are challenging to absorb and utilize by the body through cell membranes, which cannot exert biological activity [28]. Bhadja et al. [29] demonstrated that LMW polysaccharides had a greater ability to target and repair damaged renal tubular epithelial cells compared to HMW polysaccharides. This may be because LMW polysaccharides expose more reduced hydroxyl terminals, which help to scavenge free radicals and mitigate oxidative stress damage [29]. On the other hand, HMW polysaccharides may be more compact in structure, making it difficult for them to expose more active sites [30].

However, it is not the case that the lower the MW, the greater the biological activity. For fucoidan, longer sugar chains have more complex spatial conformations and more functional groups, which help it bind to multiple coagulation factors simultaneously [31].

### 3.3. Sulfation Mode

High sulfation is one of the important characteristics of MPs’ structure [1]. The amount and location of the sulfate group are critical for biological activity. Hu et al. [32] found that 4-O-sulfate substitution in fucoidan was more effective than 2-O-sulfate substitution in alleviating insulin resistance. Bhadja et al. [29] demonstrated that the antioxidant capacity of polysaccharides is directly proportional to the content of sulfate groups. Algal polysaccharides with a higher content of sulfate groups had the strongest ability to repair damaged cells. However, an excessively high degree of sulfation may also affect the spatial conformation of polysaccharides, making them difficult to be recognized by cell surface receptors [33].

Sulfated polysaccharides exhibit significant biological activity for a variety of reasons, including the following: (1) Sulfation can negatively charge polysaccharides, thereby increasing their water solubility in the appropriate MW range and thus their activity [28]. (2) The location of sulfation and epimerization on the subunits modifies the local conformation, or interactions with proteins [34]. (3) Sulfate groups interfere with the positive charge on the host cell surface through negative charge, hindering the absorption, penetration, and uncoating process of viruses [10]. (4) Sulfate groups can stimulate the anomeric carbon, increase the activity of hydrogen atoms, and thus enhance the nucleophilic properties of polysaccharides [28]. (5) Sulfate groups increase contact with immune cell receptors by combining oxygen and electrostatic attraction [28].

## 4. Biological Activities of MPs

### 4.1. Immunomodulatory Activity

**Figure 2:**
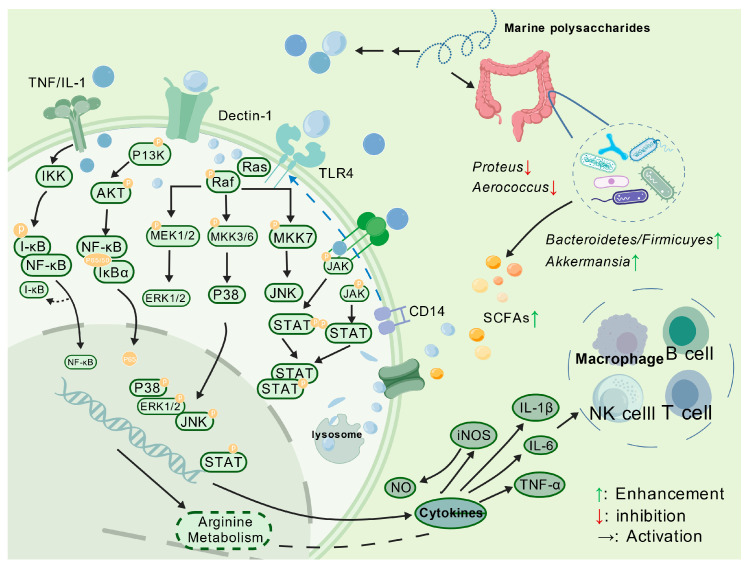
The major signaling pathways involved in the immunomodulatory effects of MPs. Created with BioGDP.com.

The purpose of the immune system is to monitor, recognize, and eliminate pathogenic and non-pathogenic microorganisms. It can conduct immune responses through the phagocytosis of macrophages, secretion of pro-inflammatory cytokines, activation of the complement system, and proliferation of immune cells. The immunomodulatory mechanisms of MPs are illustrated in Figure 2.

Khongthong et al. [35] found that polysaccharides from red algae could activate macrophages through the Dectin-1 signaling pathway, inducing NO secretion and interleukin gene expression. However, the lack of anti-inflammatory factor detection makes it impossible to determine whether polysaccharides may induce excessive inflammation. In addition to macrophages, Kim et al. [36] indicated that ulvan, extracted from *Ulva pertusa*, enhanced the activity of NK cells and lymphocytes. They pointed out that LMW ulvan after enzymatic hydrolysis demonstrated greater efficacy than the immunostimulant Krestin and HMW ulvan in activating the innate immune system. This phenomenon may be attributed to the fact that LMW polysaccharides can be more efficiently absorbed by the intestines and enter the bloodstream, while their short-chain structures may be better matched to the recognition receptors on the surface of immune cells. Sokolova et al. [37] found that carrageenan or 3,6-dehydrogalactose could activate complement C4 binding to antibodies via the classical pathway.

In addition to directly affecting the immune system, MPs can indirectly influence immune regulation by modulating the intestinal flora and short-chain fatty acids (SCFAs) metabolism. By improving intestinal flora, they promote the production of beneficial bacteria and associated metabolites, inhibit the production of harmful bacteria and related metabolites, and enhance the barrier function of the intestinal mucosa [38]. Li et al. [39] found that shellfish polysaccharides-induced alterations in gut microbiota could affect SCFA metabolism, especially butyrate metabolism. These alterations regulated gene expression in epithelial barrier cells, modulating immune responses mediated by T and B cells. However, the primary bacterial populations affected by shellfish polysaccharide, as well as the major bacterial groups responsible for elevated butyrate levels, need further investigation. Wimmer et al. [40], using 16S sequencing, found that fucoidan can enrich butyrate-producing bacteria (*Agathobacter* and *Eubacterium ventriosum*) while suppressing harmful bacteria (*Veillonella* and *Akkermansia*). Moreover, metabolomic analysis suggested that exopolysaccharides from marine coral-associated fungi might exert immunomodulatory effects by modulating amino acid synthesis and metabolism, particularly that of arginine [41]. Researchers pointed out that exopolysaccharides may promote the release of immune factors through the arginine-NO pathway. Nevertheless, the study remained at the level of metabolite associations, lacking direct evidence regarding signaling pathways like NF-κB and MAPK. Molecular targets in key pathways, such as changes in arginase and nitric oxide synthase expression, are worthy of further investigation.

Although numerous studies have provided foundational evidence for the immunomodulatory activity of MPs, significant gaps remain in in vivo validation and clinical translational relevance. Future research should integrate multiple cellular models, animal experiments, and structural modification strategies to systematically investigate the structure–activity relationships and related signaling pathways.

### 4.2. Anti-Tumor Activity

The mechanisms of tumor suppression by MPs are multifaceted. MPs have an inhibitory effect on various cancer cells such as liver cancer, ovarian cancer, lung cancer, pancreatic cancer, and melanoma [10,11,42,43].

Common anti-tumor mechanisms of MPs include inhibiting tumor angiogenesis, disrupting the cell cycle, interrupting uncontrolled cell division, and activating apoptosis pathways. Studies have shown that fucoidan can inhibit angiogenesis-related gene expression, such as VEGFs, flt1, flt4, kdr, and kdrl [43]. The inhibition of angiogenesis reduces the supply of oxygen and nutrients in cancer cells, further inhibiting their growth. It could also promote ovarian cancer cell arrest in the sub G1 phase and decreased cellular arrest in the G2/M phase, which inhibited cell growth and induced cell death in ovarian cancer cells [43]. However, Ren et al. [44] found that the anti-tumor activity of the MPs was dependent on the gut microbiota. They found that fucoidan did not affect tumor growth in pseudo-sterile tumor-bearing mice. This may because fucoidan, as a complex macromolecule, is poorly absorbed by the small intestine. Non-targeted metabolomics also demonstrated that fucoidan interfered with tryptophan metabolism through *Bifidobacterium* and *Lactobacillus*, thereby suppressing IDO1 (indoleamine 2,3-dioxygenase 1) expression in tumor tissues.

MPs also induce apoptosis through mitochondrial dysfunction and endoplasmic reticulum (ER) stress in cancer cells. Kelp polysaccharides can decrease the membrane potential of cancer cell mitochondria, increasing the production of mitochondrial Ca^2+^ and reactive oxygen species (ROS) [45]. Ca^2+^ and ROS can interact with each other, leading to mitochondrial lysis and apoptosis by extending mitochondrial membrane permeability and releasing pro-apoptotic factors [43]. Liu et al. [46] discovered that a deep-sea bacterial exopolysaccharide can trigger pyroptosis to exert anti-tumor effects. It can directly target five membrane phospholipids on the tumor cell membrane, leading to membrane rupture or pore formation. Such membrane damage may trigger K^+^ efflux and intracellular Ca^2+^ accumulation, thereby activating downstream pyroptosis signaling pathways. However, the specific dynamic changes in K^+^ efflux and Ca^2+^ accumulation, as well as whether membrane proteins (such as ion channel proteins) are involved, remain unclear.

The immunomodulatory mechanism of polysaccharides is also one of the main anti-tumor mechanisms. As previously mentioned, MPs have a wide range of immune-activating functions, contributing to the elimination of cancer cells [35]. Liu et al. [47] found that clam polysaccharides could promote the polarization of primary macrophages to the M1 type and reverse the IL-4-induced polarization of M2 macrophages toward this phenotype. It led to an increased population of anti-tumor M1 macrophages while reducing M2 macrophages that aid tumor evasion. However, the polarization induction of macrophages may also be interfered with by other factors.

### 4.3. Anti-Obesity Activity

Obesity is an unhealthy condition in which energy intake exceeds energy expenditure, resulting in an increase in fat mass and weight gain [1]. It can affect the cardiovascular, endocrine, and respiratory systems, as well as increase the stress on joints and bones and the risk of many chronic diseases [10].

MPs are considered potential sources of compounds with anti-obesity bioactivity. For example, studies have shown that MPs can alleviate diet-related obesity by modulating gut microbiota (GM). *Firmicutes* (F) and *Bacteroidetes* (B) are two of the main components of the intestinal bacterial community. A high-fat diet can increase the relative abundance of F and decrease the relative abundance of. B. Ma et al. [48] found that an oyster polysaccharide can reverse the changes in intestinal flora induced by a high-fat diet in mice, significantly ameliorating GM dysbiosis with superior effects compared to β-glucan. However, due to the wide inter-individual variation in GM, the effects of polysaccharides on flora modulation may turn out to be different among individuals.

On the other hand, MPs can inhibit fat accumulation by modulating lipid metabolism and downregulating the expression of genes related to fatty acid synthesis. Ma et al. [48] indicated that oyster polysaccharides upregulated AMPKα phosphorylation to control the binding of SREBP-1c and its downstream receptor, thus controlling cholesterol synthesis. Lee et al. [49] suggested that the fucoidan dramatically downregulated the expression levels of adipogenic peroxisome proliferator-activated receptor γ fatty acid binding protein four and lipogenic SREBP-1 proteins in 3T3-L1 adipocytes, further considerably suppressing lipid accumulation. Compared to the anti-obesity drug orlistat, this polysaccharide does not induce side effects such as diarrhea and bloating in mice. Interestingly, structural feature comparisons revealed that high-xylose-content fucoidan exhibited greater bioactivity than highly sulfated fucoidan. However, the anti-obesity activity of xylose monomers requires further validation to rule out the influence of the overall conformation of polysaccharides on their activity. In addition, Lu et al. [5] demonstrated that fucoidan could competitively bind with key amino acids in the active site of pancreatic lipase to inhibit the absorption of triglycerides.

Although extensive research has demonstrated the positive effects of MPs in combating obesity, in vitro digestion experiments cannot replicate the complex digestive environment within the body, such as gut microbiota metabolism and mucosal absorption. Future studies should further investigate the rebound of obesity phenotypes and gut microbiota following discontinuation of marine polysaccharide supplementation.

### 4.4. Anti-Inflammatory Activity

Inflammation is a defense mechanism of the body against harmful stimuli. However, persistent or uncontrolled inflammatory responses induce or exacerbate related diseases, such as arthritis, gastroenteritis, and cardiovascular disease [50].

**Figure 3:**
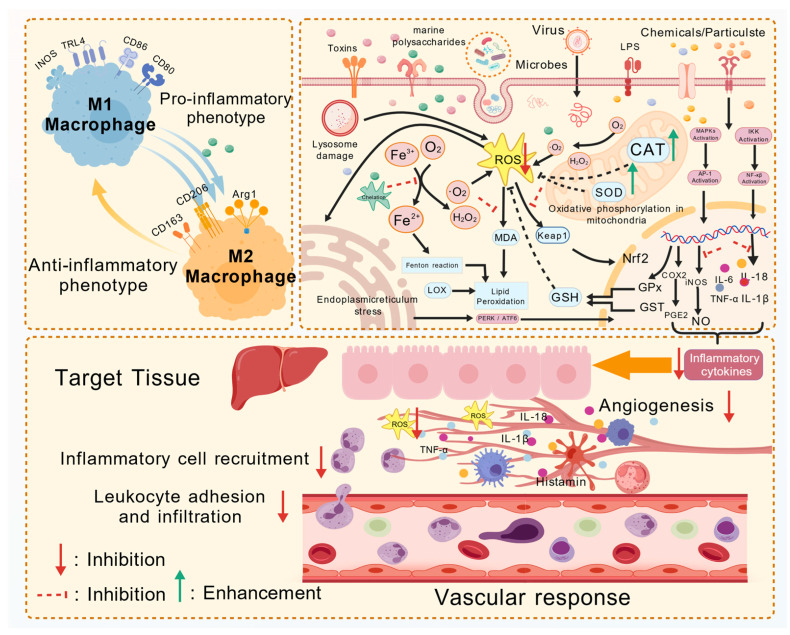
MPs exert their anti-inflammatory effect by interfering with inflammatory signaling pathways, downregulating the expression of inflammatory factors and inflammatory mediators, promoting the polarization of M1-type macrophages to M2-type macrophages, inhibiting immune cell recruitment, and suppressing the adhesion and infiltration of leukocytes. Created with BioGDP.com. Reprinted (adapted) with permission from Ref. [55]. Copyright 2025 Elsevier Ltd. (Amsterdam, The Netherlands).

MPs have been demonstrated to exhibit anti-inflammatory activity by modulating the production of inflammatory effectors and mediators involved in the inflammatory process, as shown in Figure 3. Wang et al. [8] found that sulfated polysaccharides from *Sargassum fulvellum* can restore lipopolysaccharide-stimulated macrophage viability while effectively suppressing the production of pro-inflammatory cytokines such as TNF-α, IL-1β, and IL-6. The reduction of pro-inflammatory cytokines inhibited the expression of iNOS, further reducing the production of the inflammatory mediator NO. Another study showed that fucoidan can reduce nitrite production by competing with oxygen to bind NO, acting as a chelator of NO [51]. Although results indicated that fucoidan exhibited a more pronounced NO scavenging effect than ascorbic acid, further comparative studies are warranted due to differences in their anti-inflammatory mechanisms. MPs also interfere with inflammation by regulating upstream signaling pathways, including the activation of MAPK/NF-κB signaling pathways [36]. However, Dörschmann et al. [52] investigated a HMW fucoidan from brown seaweed for potential therapeutic effects in macular degeneration models in vitro. Despite reducing levels of inflammatory cytokines (IL-6 and IL-8), FucBB04 did not provide antioxidant effects and negatively impacted key retinal pigment epithelium functions. MPs can indirectly intervene by improving intestinal structure and regulating the fermentation products of intestinal flora to maintain intestinal flora balance and immune homeostasis. Oyster polysaccharides increase levels of acetic, propionic, and butyric acids through fermentation by intestinal flora [48]. The production of such SCFAs not only supplies energy to intestinal epithelial cells but also preserves the integrity of the intestinal barrier. Shen et al. also [53] demonstrated that algal polysaccharide fermentation products significantly increased the number of beneficial bacteria and promoted the production of SCFAs. Bifidobacteria and other beneficial intestinal flora may exert effective anti-inflammatory effects on inflammatory intestinal epithelial cells by regulating TLR2-mediated NF-κB and MAPK signaling pathways.

Furthermore, polysaccharides can regulate the immune response by binding to the surface of leukocytes and inhibiting their migration to inflammatory sites. At the same time, polysaccharides with antioxidant activity can also reduce the oxidative stress associated with inflammation by scavenging free radicals [50,54].

### 4.5. Anticoagulant Activity

Normally, the clotting and anticlotting systems are in a state of dynamic balance. The imbalance between the two is the pathophysiological basis of ischemic stroke, deep vein thromboembolism, atherosclerosis, and other cardiovascular diseases.

The anticoagulant activities of MPs mainly involve inhibition of coagulation factors, augmentation of antithrombin (AT) and heparin cofactor II (HC-II), and inhibition of platelet aggregation. This is because most MPs are rich in sulfate groups, leading to the sugar chains embodying more negative charges, which in turn potentially enhances electrostatic interactions with cationic proteins such as thrombin [1].

**Figure 4:**
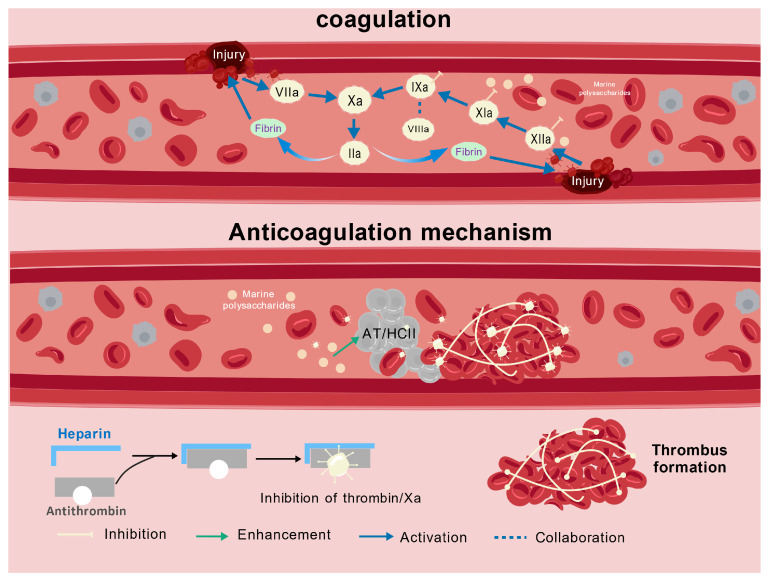
Coagulation process and anticoagulation mechanism. Created with BioGDP.com. Reprinted (adapted) with permission from Ref. [1]. Copyright 2025 Elsevier Ltd. (Amsterdam, The Netherlands).

Heparin has been widely used clinically over the years for the prevention and treatment of thrombosis. As shown in Figure 4, heparin can bind to AT-III, causing a conformational change in antithrombin, from a chronic inhibitor to a rapid inhibitor. It catalyzes the arginine center of AT to bind to the serine center of the clotting factor, inactivating the clotting factor [56]. Furthermore, heparin inhibits platelets by reducing blood viscosity and enhancing anticoagulation to minimize thrombosis [57]. However, heparin mainly targets coagulation factors in the common pathway affecting hemostasis in the coagulation cascade, which has many shortcomings and bleeding risks. In addition to heparin, other marine sulfated polysaccharides also exhibit excellent anticoagulant activity. George et al. [6] demonstrated that a sulfated galactofucan extracted from *Sargassum plagiophyllum* can simultaneously inhibit both endogenous (FXI and FIX) and extrinsic (FVII) coagulation pathways. Furthermore, this polysaccharide exhibits a sevenfold reduction in key enzyme Xa activity. Compared to traditional heparin drugs, this polysaccharide does not rely solely on its anticoagulant action. It more effectively suppresses calcium ion levels and fibrin polymerization while reducing the expression of MARCKS phosphorylation, a marker of platelet activation. Feng et al. [58] demonstrated that LMW fucoidan can specifically bind to S100A8/A9 proteins, thereby inhibiting excessive platelet activation, blocking thrombus progression, and ultimately suppressing thrombus formation. Both studies demonstrated the significant potential of marine polysaccharides in anticoagulant activity. Notably, this activity is generally correlated with the number of sulfate groups and inversely related to the molecular size [6]. Premarathna et al. [59] found that carrageenan extracted from red algae showed significantly higher anticoagulant activity than xylan due to the abundant sulfate groups. However, Maurya et al. [60] noted that moderate sulfation levels are key to the efficient binding of sulfated galactosaminoglycans to coagulation cofactors. They found that oversulfation did not improve the binding of polysaccharides to coagulation factors. More importantly, thrombus formation is often accompanied by inflammatory responses, further necessitating exploration of the synergistic effects between anticoagulation and anti-inflammation.

In summary, although numerous studies have demonstrated that the anticoagulant activity of MPs is closely associated with sulfate groups, future research may systematically elucidate the structure–activity relationship through chemical modification of molecules, thereby developing functionally oriented polysaccharide derivatives.

### 4.6. Antioxidant Activity

Antioxidant refers to the resistance to the peroxide state induced by internal cellular metabolism or external stimuli. Excessive accumulation of free radicals and ROS induces a variety of diseases, including cancer, neurological disorders, and cardiovascular disease. Many studies have demonstrated that MPs have excellent antioxidant and free radical scavenging abilities. For example, Swaminathan [61] indicated that L-fucose extracted from brown algae showed excellent DPPH, hydrogen peroxide, deoxyribose, ABTS, and superoxide radicals scavenging abilities. The free radical scavenging ability of L-fucose may be related to its sulfate groups. Compared with polysaccharides from green algae and red algae, those from brown algae may exhibit more pronounced antioxidant activity [62]. This may be attributed to the presence of more sulfated groups in fucoidan. These sulfate groups can provide electrons or hydrogen donors to react with free radicals [30]. In addition, due to hydrogen bond formation and steric effects, such groups can affect the conformation of polysaccharides, leading to the exposure of more functional groups [63]. The CS from *Sepia prashadi* cuttlebone, which contains hydroxyl and amino groups in its chemical structure, scavenges superoxide radicals and DPPH radicals and chelates ferrous iron [9]. This is because the amino group of CS can provide electrons to neutralize free radicals to clear DPPH radicals. In addition, since CS contains a relatively high number of amino and carboxyl groups with lone pairs of electrons, it can chelate Fe^2+^, forming CS-Fe^2+^ complexes [64]. MPs can additionally exert antioxidant effects by influencing relevant signaling pathways. Fucoidan has been proven to downregulate the cascade of MAPK and NF-κB signaling pathways, thereby inhibiting phosphorylation of related proteins [65]. Dörschmann et al. [66] also found that the antioxidant effects of fucoidan do not involve direct scavenging of free radicals, instead specifically regulating iron-dependent oxidative stress pathways. Wang et al. [67] found that polysaccharides from *Ulva pertusa* enhanced antioxidant enzyme activity by modulating the Keap1/Nrf2 signaling pathway. When oxidative stress occurs, Nrf2 is released from Keap1 and converted to *p*-Nrf2, which regulates the gene expression of antioxidant enzymes. This polysaccharide increased the level of Nrf2, reduced the expression of Keap1, and then increased the activities of antioxidant enzymes.

In summary, MPs have various biological activities, as shown in Table 1. However, the structure–activity relationship of MPs and their interactions with other dietary components need further investigation. Future research may innovatively combine multi-omics approaches with isotope labeling techniques to track their metabolic pathways in vivo, thereby identifying specific target molecules and elucidating the mechanisms of action and functional synergism of MPs.

## 5. Toxicity of MPs in Food

Toxicity evaluation is the premise of drug and food research and development. Although the beneficial effects of MPs in humans have been widely recognized, few studies have been conducted on their safety and potential side effects.

In vitro studies are typically designed to determine the effects of MPs on cell viability, proliferation, and morphology. The effects of the MPs on cell survival were investigated using an MTT assay in many studies. Rajan et al. [73] found that SA at 25, 50, and 75 μg/mL had no significant effect on the cytotoxicity and the morphology of SA at 25, 50, and 75 μg/mL on the viability of human umbilical vein endothelial cells. Similarly, Premarathna et al. [74] found that low concentrations of fucoidans and SA extracted from *Ecklonia maxima* were not toxic to human dermal fibroblasts cells. The toxicity of polysaccharide extracts was highly dose dependent. Cold-extracted SA did not affect the viability of RAW264.7 cells at a concentration of 0.06 μg/μL, while 0.13 μg/μL of it reduced cell viability [74]. Notably, differences in extraction methods affect the cytotoxicity of marine-derived polysaccharides. Compared to thermal extraction, low-temperature extraction better preserves macromolecules and sulfate groups but may leave residual phenolic compounds that could exhibit cytotoxicity [74]. Therefore, the purification of polysaccharides is essential. Moreover, the toxicity of MPs tends to exhibit a stronger correlation with their molecular weight than with their sulfate content. This may be attributed to the fact that polysaccharides with different MW may have different interactions with biological systems, thus showing different biological activities [74]. To sum up, these findings indicate that MPs may not be significantly toxic in vitro.

In vivo experiments are critical for validating the results of in vitro experiments. However, there is a lack of reports on the acute and chronic toxicity of MPs in vivo, which may limit their development and application. In vivo toxicity evaluation of marine-derived polysaccharides through animal experiments commonly employs intraperitoneal injection, oral administration, and aqueous exposure as typical routes of administration. Serum biochemical analysis revealed that oral administration of fucoidan at 200 mg/kg body weight (BW) to rats effectively inhibited obesity without inducing renal or hepatic toxicity [75]. However, significant variations exist in the safe dosage of marine polysaccharides from different sources. Chung et al. [76] investigated the acute toxicity of fucoidan from *Undaria pinnatifida* in rodents by oral administration for 28 days. The results showed that fucoidan at a low dose (1000 mg/kg/day) had no adverse effects on body weight, feeding, blood, or internal organs in rats. However, at a high dose of 2000 mg/kg/day, fucoidan may exhibit potential toxicity to the blood, liver, and thyroid in rats. In addition to rats, zebrafish can be employed to explore the acute toxicity of MPs. Laminarin of *Padina tetrastromatica* and *Sargassum cinereum* at 250–1000 μg/mL showed no toxic effect on zebra fish larvae [77]. In contrast, carageenan is found to be harmful to zebrafish embryos at concentrations as low as 0.4 μg/mL, causing early hatching, spinal deformities, and poor development, indicating that carrageenan may have a teratogenic risk [78]. The safety of carrageenan in the food field has aroused considerable debate. Carrageenan with MW < 100 kDa has been established as a food contaminant [79]. It may trigger inflammation, digestive problems, and immune responses. Subacute toxicity experiments and histopathology in mice also revealed that continuous oral administration of carrageenan (50–200 mg/kg) for 14 days did induce damage to the liver, kidneys, and intestines, but no significant injury was observed in the heart, spleen, or lungs [80]. In addition, carrageenan oligosaccharides cause more serious effects as they are more easily absorbed into the blood circulation, which in turn causes more serious damage to organs. Jayanthi et al. [78] demonstrated that grafting carrageenan with isoliquiritinide can mitigate its toxicity. This may be due to the fact that isoliquiritinide blocks carrageenan-induced inflammatory responses by masking sulfated groups and exerting antioxidant effects.

The final dietary fate of MPs and their degradation products in the body is unknown. The detection of marine-derived polysaccharides in vivo is a significant challenge. Most marine sulfated polysaccharides contain no UV/fluorescence chromophores, and most are not ionized in mass spectrometry. Wang et al. [80] developed a fluorescent labeling method to detect the distribution of carrageenan in mice. Pharmacokinetics showed that carrageenan (2000 kDa) is rapidly absorbed within 3 h after oral administration in mice and then remains in the bloodstream for nearly 37 h. However, due to the lack of corresponding enzymes in the human body, carrageenan cannot be degraded in the oral cavity or the intestine. Only a small amount of carrageenan can be absorbed into the bloodstream, subsequently distributing to various organs. Approximately 76.5% of the carrageenan was excreted through feces, while 9% was excreted via urine [80]. The absorbed carrageenan predominantly accumulated in the liver and kidneys of mice. This is in agreement with the results of Chen et al. [81]. Carrageenan with a MW below 2.8 kDa enters the liver and kidney of mice by oral administration. This may be because salivary glycoprotein receptors on the surface of the liver can internalize macromolecules through clathrin-mediated endocytosis and exhibit a particular affinity for galactose, leading to a higher level in the liver.

In conclusion, based on existing literature, it can be concluded that there is a lack of acute toxicity, chronic toxicity, carcinogenesis, and teratogenesis studies on MPs, with only a few studies involving toxicity studies. In particular, the specific immune responses, gastrointestinal effects, and other potential adverse effects of oral administration of MPs remain unclear. Moreover, the toxicity of MPs varies widely in vitro and in vivo. Therefore, there is an urgent need for comprehensive and systematic toxicological studies on MPs to elucidate their bioavailability, side effects, and mechanisms in vivo.

## 6. Representative Marine Polysaccharide-Based Hydrogels

MPs are common materials for hydrogels. There are numerous application examples in stimuli-responsive, self-healing, injectable, and functional hydrogels. As shown in Table 2, due to differences in physical and chemical properties, hydrogels made from MPs of different sources have different application scopes.

### 6.1. Chitosan-Based Hydrogel

CS, as a rare natural cationic polysaccharide, has become a staple raw material for intelligent hydrogels, including injectable, self-healing, and stimulus-responsive types. Owing to the presence of a substantial amount of amino and hydroxyl groups, CS can form a variety of multifunctional hydrogels in combination with other polymers [82]. For instance, under low-pH conditions, pectin and CS can form a polymer network through hydrogen bonds [83]. Additionally, given the polycationic nature of CS at acidic pH, it can form electrostatic interactions with negatively charged molecules or anions, thus improving the crosslinking of hydrogels [82]. At this pH of 3, pectin possesses a weak negative charge and could form partial electrostatic interactions with the positively charged CS [83]. However, the amino groups of CS exist in a non-ionized form in the natural environment, which limits the water solubility of CS [84]. Recently, researchers have tended to use chemically modified CS to prepare hydrogels, such as carboxymethyl CS, hydroxypropyl CS, sulfated CS, and quaternary ammonium salt CS. Quaternary ammonium CS and tannic acid can rapidly form a hydrogel through hydrogen bonding and π-π stacking [85]. Rangaraj et al. [86] utilized the isolated CS to achieve crosslinking with thiol-oxidized keratin under alkaline conditions. Thiolate anions formed in alkaline environments can initiate dynamic disulfide crosslinking reactions with thiol crosslinking agents, forming an interconnected hydrogel network. In addition, the introduction of thiol groups can also enhance the mucoadhesive properties and hemostatic activity of chitosan hydrogels [87]. Such hydrogels typically exhibit enhanced mechanical strength and encapsulation efficiency. However, trace residues may be introduced during the modification process, requiring additional purification. On the other hand, CS-based hydrogels prepared with chitosan of different MW can have considerably different characteristics depending on the interactions between the compounds [83].

Despite this, the poor mechanical strength of pure CS-based hydrogels limits its application [7]. To improve the poor mechanical properties of hydrogels, researchers tend to use dual cross-linked hydrogels, which show better strength and toughness than single-cross-linked hydrogels. Zhao et al. [88] prepared dual cross-linked hydrogels of hydroxypropyl chitosan and HA by means of Schiff base reaction and oxidative cross-linking with catechol sodium. It exhibited improved adhesion strength and mechanical strength. Notably, the catechol sodium is extracted from the adhesion component in marine mussels, which significantly improves the adhesion and waterproof properties of the hydrogel.

### 6.2. Hyaluronic Acid-Based Hydrogel

HA, as a common material for preparing hydrogel dressings, possesses excellent biocompatibility, hydrophilicity, and outstanding gel-forming properties [87]. Compared with other MPs, HA is an important component of the extracellular matrix in human tissues. It can interact with cells or growth factors, making it an attractive material for tissue engineering, especially in injectable hydrogels [13]. Nevertheless, in many cases, unmodified pure HA is not suitable for hydrogel preparation [89]. Modifiable carboxyl, hydroxyl, and amino groups on the HA molecular chains give it unique advantages in the field of novel hydrogels. For example, oxidized HA can more effectively enhance the hydrogel adhesiveness [87].

Similar to CS, HA can be converted into hydrogels under physiological conditions triggered by various stimuli, including light, temperature, pH, and ionic strength. Fan et al. [90] designed a smart responsive injectable HA-based hydrogel. Upon stimulation with low-pH conditions and glutathione, the hydrogel loaded with chemotherapeutic agents enables efficient and precise tumor chemotherapy. It can quickly cross-link in situ at the tumor site to form a porous network structure, which achieves efficient delivery and accurate release of chemotherapy drugs at the tumor site. In addition to traditional thermally responsive and pH-responsive hydrogels, researchers have developed new types of hydrogels, such as reactive oxygen species, magnetic response, and electro-responsive hydrogels [89,91,92]. However, the in situ formation process of hydrogels is difficult to monitor in vivo, limiting their safety in clinical settings. [93]. Further research is needed to determine how the hydrogel’s breakdown products affect the normal physiological functions of body tissues. Currently, research on HA-based hydrogels is primarily focused on the biomedical field, with scant research dedicated to their applications in the food industry.

### 6.3. Algal Polysaccharide-Based Hydrogel

Carrageenan, SA, and agarose are the most common hydrogel materials in algal polysaccharides. Compared with other polysaccharides, they are cheap and readily available.

Due to the unique thermal reversibility and hysteresis, agarose can form a controlled hydrogel with excellent stability, especially thermally responsive hydrogels. It can self-assemble into a non–toxic and semi-solid gel state at around room temperature [94]. Despite good mechanical properties, agar does not have any anti-bacterial activity. Hu et al. [95] developed a bacteriostatic hydrogel by embedding MXene/zinc ions in agar and SA. Such hydrogels exhibit excellent hydrophilicity, mechanical properties, and long-lasting anti-bacterial ability.

Carrageenan is another important red algal polysaccharide. Among the various types, κ-carrageenan and ι-carrageenan are the most widely used in hydrogels due to their excellent viscoelastic and gelatinous properties. They can form hydrogels and a three-dimensional double helix network through the cross-linking of neighboring sulfate groups. These gels can be thermally reversible [17]. Therefore, carrageenan is also a commonly used material in injectable hydrogels. Moncada et al. [18] developed a shear-thinning injectable hydrogel using κ-carrageenan, ι-carrageenan, locust bean gum, and gelatin as substrates. Compared with in situ gelling hydrogels, they are better for minimally invasive applications. Such hydrogels can turn into solutions by applying shear stress upon the needles and return to the hydrogel after release [93]. However, the carrageenan-gelatin system may experience gel aging due to hydrogen bond rearrangement, making it unsuitable for long-term storage. As for λ-carrageenan, the high degree of sulfation and special substitution positions endow it with extremely high electrostatic repulsion, which prevents its crosslinking and gelation [18]. Although λ-carrageenan can only form viscous solutions most of the time, it can also create firm gels in the presence of trivalent ions [17].

SA, algin gum, and laminaria polysaccharide are brown algal polysaccharides commonly used in hydrogels. SA has been widely used as a coating material in the food industry due to its excellent film-forming properties [96]. However, poor water resistance and insufficient antioxidant activity limit its application as reactive coatings [97,98]. Converting SA into hydrogel can not only improve the mechanical and waterproof properties of the packaging but also enable it to adhere more closely to the food, thus maintaining the integrity of the packaging. As one of the most commonly used materials in hydrogels, SA can rapidly create a rigid and stable SA structure hydrogel by interacting with divalent cations [99]. In recent years, SA has been widely used in stimuli-responsive, self-healing, and adhesive hydrogels and 3D printing technologies. Notably, SA can form a gel even in the presence of small amounts of metal ions. Therefore, in practical applications, it is essential to avoid rapid SA gelation, as it may hinder drug release and lead to uneven drug distribution. Another brown algal polysaccharide, fucoidan, is also commonly used in the preparation of hydrogels. However, it is usually used as an active agent rather than a substrate in hydrogels because of the high price. Similar in structure to heparin, chondroitin sulfate, and other animal polysaccharides, it can interact with various growth factors, endowing it with great potential in wound healing and tissue engineering [100].

Compared with other algal polysaccharides, due to the difficulty of processing and the lack of consistent and scalable sources, the development of green algal polysaccharides in hydrogels is relatively rare [94]. Nevertheless, green algal polysaccharides hold promising potential in hydrogels due to their good biological activity (anti-inflammatory, anti-tumor, antiviral, antioxidant, etc.). The stability of ulva hydrogel film was improved by the interaction of metal nanoparticles with ulvan functional groups. Sulastri et al. [101] successfully developed a wound excipient hydrogel by using ulvan as a substrate and adding Ag nanoparticles. In addition, it holds promising potential in controllable hydrogels due to their thermal reversibility.

### 6.4. Marine Microorganisms Polysaccharides Hydrogel

Marine microorganism polysaccharides usually contain special monosaccharides, such as fucose, GlcA, and mannuronic acid, and exhibit diversity in glycosidic linkages. However, limited by the culturing conditions and extraction rates, marine microbial polysaccharides are seldom used in hydrogels. Normally, they can only be incorporated into hydrogels as active substances.

For example, the extracellular polysaccharides of red microalgal *Porphyridium* are unique sulfated polysaccharides, which can play an immunomodulatory role by promoting immune cell proliferation and regulating cytokine activity [102]. Such extracellular polysaccharides, carrying sulfate groups and carboxylic acid groups, can crosslink with CS carrying amino groups through electrostatic interactions and hydrogen bonds. Ruiz-Davila et al. [103] exploited this crosslinking to develop a novel CS shell hydrogel, which has extremely high thermal stability, safety, adhesiveness, and drug-loading efficiency. Fillaudeau et al. [104] extracted an extracellular polysaccharide with glycosaminoglycan properties from deep-sea hydrothermal bacteria. It is rich in sulfate groups, galacturonic acid (GalA), and GlcA, and the degree of branching is extremely high. They prepared a porous heat-sensitive hydrogel by grafting it with pNIPAM. Compared with glycosaminoglycan, polysaccharides extracted from marine microorganisms are less susceptible to prion contamination.

Although marine microbial polysaccharides are unique in biological activity and structure, their development is restricted in many aspects, making it difficult to achieve commercialization. Firstly, marine microorganisms live in special environments, which have strict requirements for culturing conditions. In addition, polysaccharides extracted from different microorganisms vary a lot. The exploration, cultivation, and screening of microorganisms take a long time, and the extraction yield is unstable. The relevant laws, regulations, and supervision policies regarding marine microbial polysaccharides are imperfect, posing significant challenges in practical applications.

**Table 2:** Applications of representative marine polysaccharide-based hydrogels.

Polysaccharide	Structural/Function Components	Hydrogels Types	Crosslinking Type/Interaction Force	Features	Applications	Reference
Chitosan	Pectin; Strawberry extract	Encapsulation; Controlled release	Physical Cross-Linking	Higher stability under acidic conditions.	Functional food products	[83]
	Tannic acid	Anti-bacterial	Physical Cross-Linking	Edible; Situ rapid cross-linking	Food packaging	[85]
	Keratin; *Lacticaseibacillus rhamnosus*	Encapsulation	Chemical Cross-Linking	Encapsulate probiotics; Effective reusability	Removal mycotoxins from fruit juice	[86]
	Ag nanoparticles	Anti-bacterial	Physical Cross-Linking	Eliminate fungal; No residues	Food packaging	[105]
	Boric acid group	3D scaffold	Chemical Cross-Linking	Self-healing and reshaping capabilities; Edible	Cultured meat	[106]
	Pomegranate extract	Encapsulation	Chemical Cross-Linking	Excellent stability	Food preservation	[107]
	Ethyl cellulose; 1-methylcyclopropene	Humidity responsive	Chemical Cross-Linking	Scavenge ethylene	Food packaging	[108]
Hyaluronic acid	Anti-tumor drug	Laser; Weakly acidic; Overexpressed GSH and haase responsive	Chemical Cross-Linking	In situ forming and injectable	Tissue engineering; Cancer treatment	[90]
	Modified polyethylene glycol precursor	Electro-responsive	Chemical Cross-Linking	Promotes efficient cell migration	Wound dressing	[91]
Alginate	Agar; Zinc ion; Mxene	Anti-bacterial	Chemical Cross-Linking	Simple preparation; Synergistically acts with photothermal effect.	Wound dressing	[95]
	Curcumin liposomes; Ag nanoparticles	Reactive oxygen species; (ROS)-responsive	Chemical Cross-Linking	Antioxidant; anti-bacterial; Anti-inflammatory properties; Injectable	Diabetic wound healing	[92]
	Poly (vinyl alcohol); Mixed-dye methyl red/bromothymol blue	pH responsive	Physical Cross-Linking	Freeze resistance; Used as a sensor under a basic environment	Food Monitoring	[109]
	Phenosafranin	Encapsulation	Chemical Cross-Linking	High selectivity; High sensitivity	Detect the content of nitrite	[110]
	Gelatin; Phosphatidylcholine	3D scaffold	Physical Cross-Linking	Lower cholesterol of cultured meat	Cultured meat	[111]
	Cu^2+^; Tea tree essential oil	3D scaffold	Chemical Cross-Linking	Broad-spectrum anti-bacterial activity; Moisture responsiveness	Fruit preservation	[112]
	Chitosan; Cotton waste	Moisture absorption; Antibacterial	Chemical Cross-Linking	High water absorption rate	Food packaging	[113]
	Whey protein	3D scaffold	Chemical Cross-Linking	Cell adhesion promotion; Edible	Cultured meat	[114]
	Carbodiimide chemistries	3D scaffold	Chemical Cross-Linking	Higher mechanical properties; High cytocompatibility and cell adhesion	Cultured meat	[115]
Agar	Thiabendazole	Fluorescent sensing	Physical Cross-Linking	High sensitivity; High efficiency; excellent selectivity	Pesticide detection	[116]
	Polyvinyl alcohol; Curcumin	pH responsive	Physical Cross-Linking	Used as a sensor under an acidic environment; Higher color stability	Food Monitoring	[117]
	Konjac glucomannan	Ediable	Physical Cross-Linking	Improved springiness and chewiness; Double network	Authentic beef tripe	[118]
	Cu nanoparticles and carbon quantum dot doped with nitrogen nanocomplex	Ethylene detection	Physical Cross-Linking	Colorimetric and fluorescent responses; Highly selective	Freshness/Spoilage Monitoring	[119]
	Tributyrin	Ediable	Physical Cross-Linking	Harder; more resilient; chewier	Functional food products	[120]
Carrageenan	Konjac glucomannan	3D scaffold Allow sufficient nutrient diffusion	Physical Cross-Linking	Biocompatibility, food safety; Low cost. Support cell proliferation and allow the formation of tissue-like cell spheres	Cultured meat	[121]
	Quince seed mucilage; Red cabbage anthocyanin	Encapsulation	Chemical Cross-Linking	pH sensitive	Freshness/Spoilage Monitoring	[122]
	Alginate; Rice bran wax/soybean oil	Bigel	Physical Cross-Linking	Mimick butter; Produced shortbread	Fat substitute	[123]
Ulvan	Ag nanoparticles	Anti-bacterial	Chemical Cross-Linking	Anti-bacterial; Adsorb exudate from the wound	Wound dressing	[101]

## 7. Applications of Marine Polysaccharide-Based Hydrogels in the Food Industry

**Figure 5:**
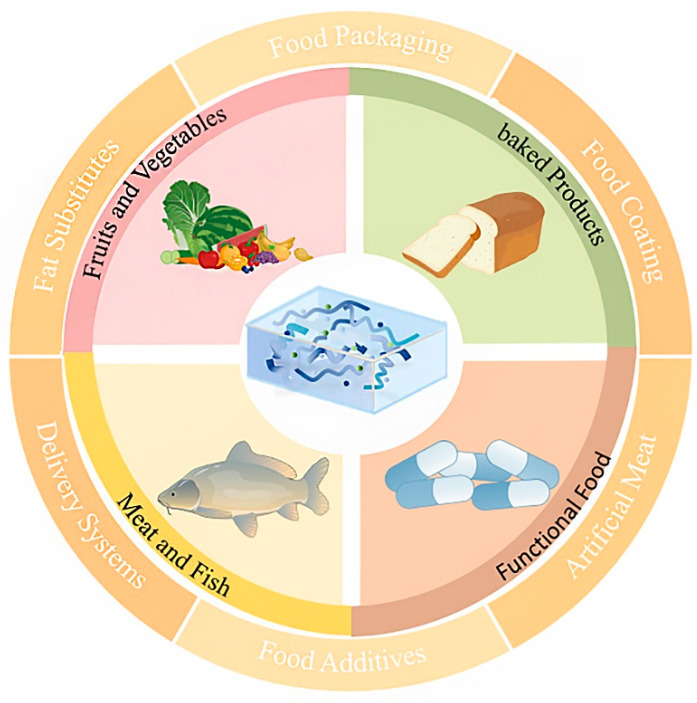
Applications of hydrogels in the food industry. Created with BioGDP.com.

MP-based hydrogels have garnered considerable attention as a promising biomaterial for various applications in the food industry. By acting as packaging coatings or films, encapsulating bioactive compounds, and constructing three-dimensional scaffolds, they have achieved substantial progress in the fields of fruits, meats, baked products, and functional foods (Figure 5). A more detailed description of their utilization in the food industry is presented in the following section.

### 7.1. Fruits and Vegetables

During the farm-to-table process, the spoilage of fruits and vegetables has led to massive waste [124]. Traditional food packaging cannot inhibit the spoilage occurring inside the package. Moreover, the poor degradability makes it harmful to the environment [125]. Owing to their excellent porosity, swelling capacity, and adsorption properties, hydrogels are well-suited as ideal carriers for antibacterial agents, thereby enabling the controlled release of these antibacterial agents [84]. Moreover, the anti-bacterial properties inherent in MPs can further inhibit spoilage [105].

Currently, CS and SA-based hydrogels have been widely applied in the development of fruit packaging materials. They are mainly used in the form of coatings, cushions, or film materials. Xie et al. [85] prepared a polysaccharide-based hydrogel by cross-linking quaternized CS with tannic acid. Compared to native CS, the quaternized form improved solubility and increased crosslinking density. This hydrogel can form a protective coating on the surface of cherry tomatoes and dragon fruit, thereby reducing moisture loss and flavor loss, and effectively maintaining the appearance of cherry tomatoes for up to 18 days. However, for fruits with delicate skins, such as strawberries, grapes, and cherries, excessive adhesion of the hydrogel may cause skin damage. Although certain MPs exhibit anti-bacterial properties, due to the constraints of microorganism type, extraction method, and chemical structure, it is necessary to introduce foreign anti-bacterial substances, such as metal nanoparticles [126], essential oils [112], and natural extracts.

Although traditional active packaging can release bioactive substances slowly, the speed and dosage are uncontrollable. Smart packaging, an innovative and multifunctional packaging approach, can control the release of active substances and perform rapid non-destructive detection to monitor food quality in real time throughout the storage, transportation, and sales chain [127]. Wu et al. [108] prepared a multi-layered film with hydrophobic ethyl cellulose as the outer layer, ethylene inhibitor and scavenger as the middle layer, and hydrogel as the inner layer. The hydrophilic inner hydrogel layer promoted the release of the ethylene scavenger by absorbing the excess moisture inside the package, thus delaying the ripening of mushrooms and extending their shelf life. Compared to monolayer films, the incorporation of hydrogel prevents functional component wastage under low humidity conditions and inhibits mold growth inside the packaging.

However, hydrogel films prepared from natural polymers are limited by their poor mechanical properties, water resistance, and ultraviolet barrier properties. Besides chemically crosslinking or modifying the hydrogel materials, researchers also prepare hydrogel beads to achieve the release of active substances. Tian et al. [112] prepared an SA-based hydrogel bead encapsulating tea tree essential oil using a metal-organic framework. As a non-contact preservative, hydrogel beads provide a new approach to the intelligent preservation of fruits and vegetables. Compared to grafting, the Cu^2+^ in MOFs can react with water molecules in high-humidity environments, thereby disrupting the structure and achieving controlled release of essential oils. In addition to preserving fruits and vegetables, MP-based hydrogels also commenced their application in the inspection of fruits and vegetables. Li et al. [128] prepared a fluorescent hydrogel sensor by incorporating Ag-modified carbon dots into an SA-based hydrogel. The highly adhesive SA hydrogel spray makes the sensor accurately and effectively detects pesticide residues. Notably, the moisture generated by fruits and vegetables during their respiration process may cause the hydrogel to soften and detach, thereby affecting the accuracy of detection.

In the field of fruit and vegetable processing, hydrogels also contribute to the improvement of product quality. The keratin-CS-based hydrogel removed mycotoxins in the fruit juice by embedding *Lactobacillus rhamnosus* [86]. This dynamic hydrogel composed of thiol-disulfide exchange provides a biocompatible carrier with favorable properties for *Lactobacillus rhamnosus,* facilitating cell adhesion and cell–cell interactions, shielding it from environmental fluctuations. Compared to the free state, encapsulated probiotics exhibit higher mycotoxin removal rates in hydrogels. However, organic acids and sugars in juices may compete for adsorption sites, potentially reducing efficiency. However, the slight astringency of chitosan may have a minor impact on the taste of the juice. Moreover, MP-based hydrogels are also expected to be applied to the clarification of fruit juice. Carboxymethyl cellulose hydrogels are reported to clarify apple juice by embedding α-amylase [15].

### 7.2. Meat and Seafood

Similar to the preservation of fruits and vegetables, MP-based hydrogels can also have great potential in the preservation of meat and seafood. For example, chitosan can serve as a coating loaded with zinc oxide and thyme oil for meat preservation [129]. Liu et al. [16] prepared an anti-bacterial CS-based hydrogel film by incorporating 3-phenylacetic acid, which effectively inactivated foodborne pathogens and extended the shelf life of chilled chicken to 4 days. Moreover, the cross-linking agent present in the hydrogel network, such as glycerol, can bind water molecules through hydrogen-bond interactions, inhibit the formation of ice crystals, and improve the sensory quality of frozen chicken [19].

During the preservation, some MP-based hydrogels can not only play a role in preservation but also monitor food freshness. Luo et al. [19] developed a CS-based hydrogel for tracking the freshness of chicken breast. It can effectively load pH-sensitive pigments. The release of volatile basic nitrogen compounds such as ammonia and amines creates an alkaline environment inside the packaging, inducing color changes in the indicator. pH-sensitive hydrogels have superiority in enhancing color response sensitivity, due to their high hydrophilicity and water-holding capacity, facilitating the acid–base reaction of volatile gas [109]. However, the impact of humidity and oxygen concentration in meat storage on color response has not yet been investigated. Jang et al. [130] successfully employed SA hydrogel microspheres loaded with poly (diacetylene) to detect bio-ammonia formation in pork. In comparison to the former, these microspheres are easy to rupture under compression, making them more suitable for static, non-interference scenarios. In addition to freshness detection, hydrogels have also begun to be applied to the detection of food additives. SA-based hydrogel enhances the sensitivity between nitrite and probe molecules through the efficient encapsulation and dispersion of Au nanoparticles [110].

In recent years, traditional livestock farming has been under pressure from resource and environmental sustainability [131]. Cultured meat, derived from culturing cells in vitro rather than slaughtering livestock, is intended to supply a stable, sustainable, and ethical meat product [111]. Hydrogels have been discovered as ideal biological scaffolds for cultured meat. Edible and safe cell-cultured meat scaffolds are the core points. Due to the complex and multicellular nature of meat culturing, the scaffold structure should be three-dimensional, which can provide structural support to the cell dynamic behavior [115]. Hydrogels with a three-dimensional cross-linked network structure could not only allow sufficient nutrient diffusion, protecting cultures from high shear stress, but also promote the directional growth and differentiation of muscle stem cells [121]. Moreover, the high water content of hydrogels is a key contributor to their ability to simulate the texture and consistency of natural meat.

SA, agar, and carrageenan have been investigated for cultured meat, due to their biocompatibility, low toxicity, low cost, and mild gelation. Moreover, the tunable mechanical and physical properties can be well-tailored to cellular requirements. For example, SA-based hydrogel can support the proliferation and differentiation of porcine muscle stem cells [132]. However, natural SA-based hydrogels exhibit a deficiency in cell adhesion and diffusion [115]. Muscle stem cells experienced a rapid decline in their adhesion, proliferation, and differentiation capabilities following short-term in vitro culture [132]. During the long-term culture of cultured meat, changes in scaffold properties may affect cell growth. To overcome the limitations of SA hydrogels, Melzener et al. [133] incorporated zein fibers into the hydrogel matrix, thereby accelerating compaction and enhancing cell-gel interactions. The incorporation of zein fibers disrupted the ionic cross-linking between SA and Ca^2+^, making the hydrogel more susceptible to degradation by enzymes secreted by cells or environmental factors. This may create space for cell spreading and tissue compression. However, the cellular metabolic activities, nutrient transport, and extracellular matrix secretion within the SA-hydrogel scaffold are unclear. Park et al. [134] have applied non-targeted metabolomics to compare cultured meat with conventional chicken, revealing differences in numerous amino acid metabolic pathways. This may result in variations in human nutrient absorption. κ-Carrageenan/konjac glucomannan hydrogels can provide mechanical support for porcine subcutaneous pre-adipocytes and obtain structured cultured fat by adipogenic differentiation for the development of cultured “snowflake pork” [121]. The addition of carrageenan can create smaller, uniform network cavities in the gel and improve its hardness and chewiness. Additionally, long-term usage can lead to the partial dissolution of the hydrogel, preventing the formation of larger fat droplets. To better mimic the texture and mouthfeel of meat, hydrogels also need to support the co-differentiation of multiple cell types simultaneously. However, this study only focused on the aggregation of fat cells, without verifying whether fat cells cultured in 3D hydrogels can be co-cultured with muscle cells to form a spatial distribution similar to that of natural “snowflake meat.”

Although hydrogels exhibit great potential for cultured meat production, polysaccharide-based hydrogels still face numerous challenges. Poor mechanical properties are the core bottleneck. Inherent low stiffness and high brittleness make it difficult to match the physiological mechanical environment of different meat tissues. Meanwhile, low cell adhesion efficiency limits cell engraftment. Existing solutions include chemical modification or complexation with biomolecules to provide additional attachment sites [115,135]. Moreover, it is difficult to control the uniform distribution of nutrients in the hydrogel, which potentially leads to unbalanced nutrient uptake by cells, thus affecting the quality of cultured meat. While current research on hydrogel-based cultured meat mainly focuses on poultry and livestock meats, research on fish meat is extremely scarce.

Plant-based meat is another emerging research area for hydrogels. Traditional plant-based meat, composed of single tissue proteins, lacks the hierarchical structures inherent in genuine meat products. Moreover, it suffers from a notable deficiency in flavor [136]. Hydrogel, endowed with preeminent encapsulation capabilities and distinctive physical attributes, can compensate for the shortcomings of traditional plant-based meats, such as flavor, taste, and texture. Du et al. [118] prepared an agarose/konjac glucomannan hydrogel by heating-cooling combined with a sodium carbonate immersion strategy. This hydrogel with excellent mechanical strength and toughness can mimic the texture of beef tripe. Liu et al. [136] successfully simulated the connective tissues of animal muscles using algal-derived polysaccharide hydrogels. They can improve the organoleptic diversity of plant-based meat. Furthermore, the unique hydrogel structure can also endow plant-based meat with an odor-release mechanism similar to that of traditional meat by controlling the release of odor molecules [137]. For the development of new products such as plant-based meat and cultured meat, hydrogels are a very promising manufacturing technology, yet there is still ample room for improvement.

Overall, MP-based hydrogels have attracted extensive attention in the meat product field due to their excellent properties. However, there are still some deficiencies that need to be improved, especially in terms of long-term stability, specificity, structural design, and interactions with nutritional components, which require further research and verification.

### 7.3. Baked Products

With the progress of the modern food industry, solid fats such as butter and hydrogenated vegetable oils have been extensively utilized in baked foods [123,138]. However, traditional solid fats contain high levels of saturated fatty acids or trans-fatty acids, which are likely to induce chronic illnesses such as obesity, atherosclerosis, and cardiovascular disease [123]. The reduction of saturated fatty acids in the food industry has become an important issue in recent years. Bigels, a novel two-phase system made of hydrogels and oleogels, have great potential in the development of fat substitutes. They entrap both oil and water phases in a three-dimensional network [138]. Researchers have applied this system to the development of low-fat biscuits, bread, and cakes. Nutter et al. [123] investigated a partial replacement of butter and shortening in shortbread by bigels, which are composed of an SA/κ-carrageenan-based hydrogel and a rice bran wax/soybean oil oleogel. The physicochemical analyses showed that compared with traditional shortbread, bigels did not significantly affect cellular structure or moisture content. The addition of hydrogel prevented the cookies from becoming overly dry and provided internal support.

Moreover, studies have shown that the embedding effect of hydrogels can also be used to improve the sensory, nutritional, and anti-bacterial properties of baked products. Yeasts encapsulated in SA hydrogels can improve the sensory characteristics of frozen dough bread [20]. During the frozen storage, SA, which has film-forming properties, can slow down the heat transfer within the hydrogel and overcome the low-temperature sensitivity of baker’s yeast. Moreover, the bread containing hydrogels had better color, flavor, and aroma than the bread without hydrogels during a consumer evaluation. Another study showed that the incorporation of CS into the dough achieves anti-glycation performance [139]. CS can inhibit the oxidation of proteins into advanced glycation end products by competing with reducing sugars for amino acids. Compared to the hydrogel in bigels, this study primarily focuses on the dynamic interactions between hydrophilic groups such as hydroxyl and amino groups in chitosan and the water and proteins within the material. Although the authors proposed that chitosan can inhibit advanced glycation end products by reacting with proteins and competing for reducing sugars, it lacks in-depth research. Similarly, when hydrogels are fabricated into active packaging, they can be applied to the preservation of baked products [140].

### 7.4. Functional Food

One primary principle in the design of functional food matrices involves the encapsulation, protection, and controlled release of nutrients. Most bioactive substances, such as polyphenols, fatty acids, ferulic acid, and probiotics, are sensitive to environmental stimuli, including oxygen, temperature, pH, and excessive metal ions, which limits their effectiveness [120,141,142]. One of the main applications of food hydrogels is the encapsulation of such bioactive molecules. The encapsulation of bioactive ingredients within a hydrogel network can improve the ingredient’s stability, bioavailability, and bioactivity.

Alginate and CS-based hydrogels are gaining increasing popularity as encapsulating agents due to their excellent encapsulation efficiency, biocompatibility, low cost, and environmental friendliness. Researchers have successfully encapsulated strawberry polyphenols, using pectin and CS-based hydrogels [83]. Under acidic conditions, the amino groups of CS are more protonated, which in turn makes the hydrogel swell. Such encapsulation of sensitive bioactive compounds can delay their degradation in the oral cavity or stomach, enabling their successful delivery to the intestines, maximizing the potential for human absorption. Honey encapsulated with alginate also proved to exhibit less than 20% bioavailability during gastric digestion [143]. However, different phenolic compounds require specific polysaccharides for encapsulation. As an anionic polysaccharide, alginate exhibits non-protonated properties, the charge state of which does not change significantly with pH. Thus, it is more efficient at encapsulating cationic active substances like anthocyanins. Conversely, chitosan, with its -NH_3_^+^, is better suited for encapsulating anionic active substances such as phenolic acids.

In addition to compounds, hydrogels can also be used to encapsulate microorganisms. Lasta et al. [141] employed alginate hydrogel to successfully encapsulate *Lactobacillus acidophilus*. Under acidic conditions, the carboxylic acid groups of alginate are protonated (existing as -COOH groups). By forming hydrogen bonds with hydroxyl groups, the hydrogel shrinks, slowing down the probiotic release. Subsequently, they incorporated the encapsulated product into chocolate coatings. This innovative application effectively shielded the probiotic cells from the adverse effects of the gastric and intestinal environments. In addition, probiotics are capable of producing organic acids (lactic acid), affecting microbial stability, shelf life, and sensory evaluation [144]. Recently, some studies have been dedicated to the preparation of pH-responsive hydrogels, which aim to retain more drugs in the gastric environment. When these hydrogels are orally administered, they rapidly swell in the acidic gastric juice, preventing their discharge into the small intestine and thus prolonging the drug release time in the stomach [145]. Sun et al. [120] developed agar hydrogel beads encapsulating tributyrin, which hold promise for replacing tapioca pearls in bubble tea. Compared with tapioca-based boba beads, agar-based boba beads exhibit better elasticity and chewiness. They are also less sensitive to changes in the gastrointestinal tract environment, which helps to improve the bioaccessibility of tributyrin. Overall, MP-based hydrogels are ideal carriers for active substances. This property is highly effective in enhancing the bioaccessibility of nutrients and functional components, holding great potential for applications in the functional food industry.

### 7.5. Biocatalyst

The process of biocatalysis has been immensely used in various sectors of the food industry because of their high substrate specificity, high chemo-selectivity, and green chemistry. However, limited by reusability, stability, and loss of activity, it is expensive to use enzymes in biocatalysis [146]. Therefore, immobilized enzymes were developed too against diverse conditions. Immobilization significantly improves catalytic efficiency by preventing aggregation and imparting a rigid structure, thus preserving enzyme activity [147]. MP-based hydrogel is a promising enzyme immobilization material due to its biodegradability, biocompatibility, and inert and hydrophilic nature. The highly porous structure of hydrogels reduces limitations of substrate transfer through the matrix of hydrogel. The aqueous environment of it minimizes enzyme denaturation and maintains the catalytic function of enzymes [148]. Common methods of enzyme immobilization include adsorption, encapsulation, entrapment, covalent attachment, and cross-linking, which have been thoroughly summarized by Leila et al. [149].

MP-based hydrogels such as chitosan, carrageenan, alginate, hyaluronic acid, etc. have been used as supports for enzyme immobilization. For instance, SA and gelatin as a wall material and glutaraldehyde as a cross-linking agent were used to form hydrogels for the encapsulation of lipase enzyme [150]. Notably, SA with a porous structure could not be used alone because gastric acid may easily enter into the inner of SA, resulting in the inactivation or degradation of the core [151]. The hydrogel can operate for six consecutive cycles while maintaining a highly active state, and the initial activity of immobilized lipase was 90% after 30 days. The long-term storage stability of the immobilized enzyme proved suitable compatibility between the hydrogel and the entrapped enzyme [147]. Moreover, due to the porous structure of the hydrogel, the as-produced free fatty acid could diffuse into the bulk solution, enabling concurrent production and delivery of fatty acids. However, glutaraldehyde, as a hazardous substance, may have residues that irritate the human respiratory system, skin, and eyes [152]. The presence of the denatured enzyme may also hinder active enzymes from diffusing at the interface. It has been shown that enzymes can be immobilized on supports having opposite ionic nature under the same conditions. Due to the abundance of SO_3_^−^ groups in structure, MPS-based hydrogels exhibit unique advantages in this method. D’Almeida et al. [153] created a relatively rigid hydrogel bead on the interaction between Al^3+^ and SO_3_^−^ groups of ι-carrageenan. The large pore diameters obtained during the formation of hydrogel beads may permit the enzymes immobilized in this support. By adjusting the pH, the particle size of the hydrogel beads was altered. However, such immobilized enzymes are easily disrupted by pH changes, as the electrostatic repulsion of SO_3_^−^ under alkali conditions leads to carrier instability [154].

MP-based hydrogels have been applied to the immobilization of several enzymes, such as protease [155], lipase [150], cellulase [156], and β-galactosidase [157]. The benefits of MP-based hydrogel for enzyme immobilization include simplicity, ease of handling, reusability, efficiency, and affordability. However, the poor stability of natural hydrogels may lead to enzyme leakage during processing. In existing studies, the relationship between the structural characteristics of MPs and enzyme immobilization efficiency and catalytic performance remains unclear. For instance, the impact of chitosan hydrogels with different degrees of acetylation on enzyme binding sites and catalytic efficiency has not been clarified.

## 8. Conclusions and Future Prospects

As one of the major resources of marine organisms, MPs have been intensively studied for years. Unique structure, biological activity, and biodegradability make them ideal materials for hydrogels. The abundance of modifiable functional groups in their molecular structures offers great potential for precisely regulating hydrogel properties. These properties give them high hydrophilicity, controllable swelling characteristics, and the ability to encapsulate and release active substances, demonstrating broad application prospects in the food industry. Functional hydrogels and stimuli-responsive hydrogels have wide applications in food packaging and detection. The controllable swelling behavior and controlled digestibility can ensure the effective delivery and sustained release of nutrients and probiotics. In addition, owing to their excellent hydrophilicity, water-holding capacity, and biocompatibility, MP-based hydrogels also have promising application prospects in emerging fields such as cultured meat and plant-based meat, thereby contributing to technological innovation and product optimization in these fields.

However, despite the significant potential of MP-based hydrogels as novel green encapsulation systems in the food industry, several challenges need to be resolved before MP-based hydrogels are truly introduced into production. One of the primary limitations lies in the alteration of mechanical integrity. Structural modification of marine polysaccharides is a common approach to enhance their performance. Such structural changes inevitably lead to alterations in biological activity. Despite the fact that most studies have clearly demonstrated that parameters such as sulfation degree and molecular weight affect biological activity, few papers have explored the impact of modifying specific structural units (e.g., glycosidic bond type, branching degree) on the quantitative regulation mechanisms of hydrogels. This limitation also constrains function-oriented design. Future research directions should focus on developing sophisticated controlled-release mechanisms, improving encapsulation efficiency, and improving mechanical strength. In addition, the industrial application of MP-based hydrogels also faces key bottlenecks associated with material safety and mechanism analysis, including the following:

(1) Increase in toxicological studies: Most of the documented bioactivities of MPs are still in the research stage. However, the destruction of marine ecosystems leading to marine organisms (especially algae) can enrich pollutants, such as heavy metals and polycyclic aromatic hydrocarbons. Consequently, the absence of clinical trials, especially toxicological studies including acute toxicity, chronic toxicity, and teratogenicity testing, constitutes a key constraint on the practical application of marine polysaccharides.
(2) Drawbacks of natural polymer hydrogels: Poor mechanical properties, insufficient long-term stability, and weak anti-interference ability of natural polymer hydrogels are key factors limiting their applications. Therefore, the development of safe and efficient cross-linking technologies is urgently required to improve their performance.
(3) Modern computational methods: Based on extensive structural and activity data of marine polysaccharides, artificial intelligence (AI), bioinformatics, and chemoinformatics can contribute to predicting the structure and function of MPs, analyzing the interactions between polysaccharides and proteins/lipids, simulating the interactions between MPs and biological targets, and further screening target polysaccharides.
(4) Multi-omics: Multi-omics integration (e.g., proteomics, metabolomics, and genomics) can better elucidate the metabolic pathways and interactions between hydrogels and bioactive components in vivo, which will facilitate delving into the underlying molecular mechanisms.

## Figures and Tables

**Table 1:** Composition, structural characteristics, and bioactivities of MPs. ↓ denotes inhibition or reduction; ↑ denotes enhancement or activation.

Polysaccharides	Source/Species	Main Monosaccharide	Backbone	Substitution Position of Sulfate Group	Biological Activity	Structural Features	References
Sulfated Fucoidan	*Durvillaea antarctica* (Brown Alga)	L-Fucose, Xylose, Galactose, Mannose	(1→3)-α-L-Fucose, (1→4)-α-L-Fucose	C-4((1→3)-Fucose), C-2((1→4)-F Fucose), C-6((1→4)-Galactose)	Immunomodulatory activity (macrophage/NK cell activation, lymphocyte↑, NO↑)	Highly sulfated (>20%), no glucose, terminal residues (β-D-Xylp-(1→) and (β-D-Galp-(1→)	[68]
Sulfated Fucoidan	*Fucus vesiculosus* (Brown Alga)	L-Fucose	α-(1→3)-L-Fucose, α-(1→4)-L-Fucose	C-2/C-4 positions of Fucose	Anti-ovarian cancer activity (sub-G1 phase↓, caspase-3/9↑, PI3K/Akt↓, MAPK↓, Anti-angiogenesis)	Highly sulfated	[43]
Sulfated Fucoidan	*Laminaria japonica* (kelp)	L-Fucose, D-Galactose, D-Mannose	—	C-4 position of Fucose	Anti-obesity activity (pancreatic lipase↓)	Highly sulfated (>25%), negatively charged, high digestive resistanc	[5]
Laminarin	*Laminaria digitata*	D-Glucose	β-(1→3)-D-Glucos, β-(1→6)-linked glucose branches		Anti-ovarian cancer activity (cell cycle arrest, mitochondrial function, apoptosis↑, Ca^2+^ homeostasis, PI3K/Akt↓, MAPK↓)	LMW, single monosaccharide, lacking modified groups	[45]
Sulfated Galactan,	*Gracilaria fisheri* (Red Alga)	D-Galactose, D-Glucose, D-Xylose	1,3-β-D-Galactopyranose, 1,4-α-L-anhydrogalactopyranose	C-6 position of 1,4-α-L-galactose	Immunomodulatory activity (Pro-Inflammatory Cytokine↑, iNOS↑, Dectin-1↑, Macrophage activity↑)	High galactose purity, highly sulfated, HMW, O-methylation	[35]
	*Enteromorpha prolifera*	Rhamnose, Glucuronic acid	α- and β-(1,4)-linked monosaccharides	C-3 position of rhamnose	Immunomodulatory activity (pro-inflammatory cytokines, Caecal microbiota modulation)	LMW	[38]
	*Patinopecten yessoensis* (Scallop)	—	—	—	Immunostimulatory activity (Immunoglobulin↑) *Bacteroides/Firmicutes*↑, TLR↑)	No sulfate groups, not hydrolyzed by human digestive enzymes, prebiotic function	[48]
Sulfated Polysaccharide	*Sargassum fulvellum* (Brown Alga)	L-Fucose, D-Galactose, D-Galactose, D-Xylose	—	—	Anti-inflammatory activity (iNOS↓, COX-2↓, Pro-inflammatory cytokines↓)	Moderately sulfated (>1%)	[8]
Ulvan	*Ulva linza* (Green Alga)	L-Rhamnose, D-Glucuronic Acid	β-D-GluA-(1→4)-α-L-Rhamnose, α-L-IdoA-(1→4)-α-L-Rha3S	C-3 of L-Rhamnose	Antioxidant activity (DPPH↓, ABTS↓, ROS↓, Microbiota Modulation, SCFAs↑)	High-content rhamnose and glucuronic acid, HMW	[69]
Extracellular Polysaccharide	*Limosilactobacillus*	Glucose, Mannose, Galactose	—	—	Antioxidant activity (DPPH↓) Anti-inflammatory Activity (iNOS↓, COX-2↓) Immunomodulatory activity (NO↑, IL-6↑, TNF-α↑)	LMW, minor glucuronic acid and galacturonic acid	[70]
Sulfated Chondroitin	*Phyllophorella kohkutiensis* (Sea Cucumber)	D-Glucuronic Acid, D-Galactose, D-Glucosamine, N-Acetylgalactosamine, L-Fucose	β-D-Glucuronic Acid-(1→3)-β-D-N-Acetylgalactosamine	C-4 position of Fucose, C-2 /C-4 position of Fucose, C-6 position of N-Acetylgalactosamine	Antioxidant activity (DPPH↓, ABTS↓, FRAR↑)	High-content fucose, moderate sulfated (>10%)	[71]
Aminopolysaccharide	*Agelas aff. Nemoechinata*	Mannose, N-Acetylglucosamine, N-Acetylgalactosamine, Galactose, Fucose	α-(1→2)-Mannose/α-(1→6)-N-Acetylgalactosamine	—	Anti-liver cancer activity (MAPK/mTOR/TNF/Hippo, umor angiogenesis↓, Mitochondrial apoptosis pathway↑, Migration and invasion↓)	HMW, high-Content aminoglycoside, multi-type sidechains	
Oxidized Fucoidan	Brown algal	Fucose	(1→3)-α-Fucose, (1→4)-α--Fucose		Antioxidant activity (ROS↓, Pro-inflammatory cytokines↓)	Aldehyde group	[72]
Carboxymethyl Chitosan	Crustaceans	Glucosamine	β-(1→4)-Glucosamine		Mucosal adhesion↑	C-6 hydroxylation or amination with carboxymethylation	
Sulfated Galactan	*Botryocladia occidentalis*	D-Galactose, 3, 6-Anhydrogalactose	α-(1→4)-galactose, β-(1→3) galactose	C-2 or C4 positions of Galactose	Antioxidant activity (binding to heparin cofactor II)	Low sulfation, methylation	[60]

## Data Availability

Not applicable.

## Abbreviations

The following abbreviations are used in this manuscript:

MPs	marine-derived polysaccharides
CS	chitosan
HA	hyaluronic acid
Glc	glucose
Gal	galactose
Man	mannose
Fuc	fucose
GalA	galacturonic acid
GlcA	glucuronic acid
IdoA	iduronic acid
LMW	low molecular weight
HMW	high molecular weight
APTT	activated partial thromboplastin time
TT	thrombin time
VEGF	vascular endothelial growth factors
IL-1/6/10	interleukin-1/6/10
IL-1β	interleukin—1Β
TNF-α	tumor necrosis factor-A
DPPH	2,2-diphenyl-1-picrylhydrazyl
ABTS	2,2’-azinobis-(3-ethylbenzthiazoline-6-sulphonate)
CAT	catalase
SOD	superoxide dismutase
GSH-PX	glutathione peroxidase
Bax/Bcl	Bcl2-associated X
mTOR	mammalian target of rapamycin
NF-κB	nuclear factor kappa-B
TLR2/3/4	toll-like receptor 2/4
SCFAs	short-chain fatty acids
Dectin-1	dendritic cell-associated C-type lectin receptor
SR	scavenger receptor
ERK 1/2	extracellular-regulated kinase 1/2
p38	P38 mitogen-activated protein kinase
JNK	C-Jun N-terminal kinase
JAK2	Janus kinase
PI3K-AKT	phosphatidylinositol 3-kinase/Akt
ER	endoplasmic reticulum
ROS	reactive oxygen species
GM	gut microbiota
MAPK	mitogen-activated protein kinase
JAK-STAT	The Janus kinase-signal transducer and activator of transcription
NF-κb	nuclear factor-kappa B
AT	antithrombin
HC-II	heparin cofactor II
SA	sodium alginate
